# Individual and neighborhood social determinants and high-risk behaviors among Puerto Rican young adults: A cross-sectional analysis of the PR-OUTLOOK Study

**DOI:** 10.21203/rs.3.rs-10832018/v1

**Published:** 2026-09-28

**Authors:** Mary R. Lee, Cynthia M. Pérez, Milagros C. Rosal, Sarah N. Forrester

**Affiliations:** University of Massachusetts Chan Medical School; University of Puerto Rico, Medical Sciences Campus; University of Massachusetts Chan Medical School; University of Massachusetts Chan Medical School

**Keywords:** Puerto Ricans, young adults, Social determinants of health, social vulnerability, health behaviors, cigarette smoking, e-cigarette use, recreational marijuana use, binge drinking, physical inactivity

## Abstract

**Background:**

Health-risk behaviors, including tobacco use, excessive alcohol consumption, and physical inactivity, contribute to chronic disease and are shaped by individual and neighborhood social conditions. However, little is known about how individual social disadvantage and neighborhood social vulnerability relate to health-risk behaviors among young adults residing in Puerto Rico. The objectives of this study were to examine associations between individual- and neighborhood-level social determinants of health and engagement in high-risk behaviors among Puerto Rican young adults residing in Puerto Rico.

**Methods:**

We analyzed baseline data (2020–2024) from the PR-OUTLOOK cohort, including 2,727 adults aged 18–29 years residing in Puerto Rico. Individual-level social determinants of health were analyzed using a latent factor score derived from ten indicators and categorized into tertiles (least, moderate, and most disadvantaged). Neighborhood-level social vulnerability was assessed using the census-tract level Social Vulnerability Index (continuous range: 0–1.0). Five binary high-risk behavior outcomes were analyzed: cigarette smoking, e-cigarette use, recreational marijuana use, binge drinking, and physical inactivity. Poisson regression with robust standard errors was used to estimate adjusted prevalence ratios (aPRs) for individual-level social determinant groups; models were subsequently stratified by sex to assess sex-specific associations. Fixed-effects logistic regression with cluster-robust standard errors at the census-tract level was used to estimate adjusted odds ratios (aORs) for individual-level social determinants of health and neighborhood-level social vulnerability.

**Results:**

Among participants, 33.7% reported one high-risk behavior, 25.1% reported two, and 18.5% reported three or more. Compared with the least disadvantaged group, individuals in the most disadvantaged social determinants of health tertile had a higher prevalence of cigarette smoking (aPR: 1.71) and physical inactivity (aPR: 1.24), with stronger and more consistent associations observed among female participants. At the neighborhood-level, higher social vulnerability score was associated with increased odds of binge drinking (aOR: 1.85) but lower odds of e-cigarette and marijuana use.

**Conclusions:**

In this cohort of Puerto Rican young adults, both individual-level and neighborhood-level social disadvantage were associated with selected high-risk health behaviors, particularly among female participants. Future longitudinal studies are needed to elucidate causal pathways and inform multi-level interventions aimed at reducing disparities in high-risk health behaviors in this population.

## Background

Health-related risk behaviors such as tobacco use, excessive alcohol consumption, and physical inactivity contribute to chronic diseases, including cardiovascular disease (CVD) and cancer. Social determinants of health (SDOH), such as economic stability, housing, education, and healthcare access, shape individuals’ engagement in these behaviors [1]. SDOH creates opportunities, constraints, and stressors that influence behavioral choices and risks [2–6]. Living in deprived or socioeconomically disadvantaged neighborhoods is generally associated with poorer health outcomes and a higher incidence of CVD [1, 6]. However, the strength and direction of these associations often vary across population subgroups and geographical contexts.

In Puerto Rico, the longstanding economic instability, austerity-driven public policies, efforts to recover from natural disasters (notably Hurricanes Irma and María in 2017) [7–9], and the COVID-19 pandemic have intensified socioeconomic hardship and heightened social vulnerability [10, 11]. These conditions may contribute to elevated stress and reduced access to supportive environments, potentially contributing to high-risk behavior engagement among young adults [12]. Compared with other United States (U.S.) Hispanic/Latino groups, including Mexican-Americans and Cubans, Puerto Rican adults living in the continental U.S. have exhibited higher prevalence of health-related risk factors such as smoking, excessive alcohol use, and physical inactivity [13]. Prior research has reported elevated prevalence of cigarette smoking, alcohol consumption, and physical inactivity among Puerto Rican adults [3, 13–15], with patterns often shaped by socioeconomic gradients [16]. However, relatively little is known about Puerto Ricans and young adults living on the island of Puerto Rico.

Importantly, previous studies have reached mixed conclusions regarding whether individual- or neighborhood-level disadvantage plays a more dominant role in influencing health behaviors among Puerto Ricans [8, 13, 14, 17–19]. Some evidence points to stronger associations with individual-level factors and socioeconomic hardships [13, 14, 17, 18], whereas others emphasize the importance of contextual disadvantage, acculturation patterns, and disaster-related stress exposure as key drivers of high-risk behaviors [8, 19]. These inconsistencies reflect broader uncertainty about the mechanisms linking social context to health-related risk behaviors across diverse Latino populations.

Although national surveillance systems such as the Behavioral Risk Factor Surveillance System (BRFSS) include Puerto Rico and provide age-stratified estimates, public data are not readily linkable to neighborhood-level indicators, limiting their utility in examining context-specific behavior risks during young adulthood [20]. In addition, studies of Puerto Rican populations in the mainland U.S. have also shown poorer cardiometabolic profiles and health behaviors compared with other Latino groups [15, 21–23], but these populations tend to experience greater social disadvantage than the general U.S. population. In Puerto Rico, prior studies have documented the high prevalence of cardiometabolic conditions and associated health behaviors among adults. Findings from Puerto Rico BRFSS and other local cohort studies have shown elevated prevalence of smoking, alcohol use, obesity, hypertension, and diabetes, particularly among individuals with lower socioeconomic status and females [14, 15, 22, 24, 25]. However, most of this research has focused on midlife and older adults, limiting insight into high-risk health behavior patterns during young adulthood.

There is a dearth of epidemiological studies examining how both individual- and neighborhood-level SDOH jointly shape high-risk health behavior engagement among Puerto Rican young adults residing in Puerto Rico. Given the developmental significance of young adulthood and the island’s current social and economic conditions, this population may be vulnerable to structural influences on health behavior. High-risk health behaviors such as smoking, excessive alcohol use, and physical inactivity often co-occur, driven by shared social, environmental, and psychological determinants [25]. Examining multiple behaviors simultaneously may help reveal underlying mechanisms and identify points for future intervention. Additionally, previous research suggests that SDOH may operate differently across sexes, particularly in settings with a greater presence of structural inequities [26, 27].

## Methods

Accordingly, this study sought to characterize individual-level SDOH and high-risk behaviors among young adults in Puerto Rico, assess associations between individual-level SDOH and high-risk behaviors, and examine whether neighborhood-level disadvantage, measured using the Social Vulnerability Index (SVI), is associated with the likelihood of engaging in high-risk behaviors. We hypothesized that both individual and contextual SDOH contribute to the prevalence of high-risk behaviors among young adults in Puerto Rico.

### Study design and setting

Data were obtained from the Puerto Rico Young Adults’ Stress, Contextual, Behavioral & Cardiometabolic Risk (PR-OUTLOOK) study, a cohort study initiated in September 16, 2020, with in-person baseline data collection through December 31, 2023 [28, 29]. The cohort consists of 3,050 young adults aged 18–29 years residing in Puerto Rico, designed to investigate cardiovascular health and CVD risk factors in this population. Recruitment occurred through community outreach, media campaigns, and participant referrals; detailed recruitment procedures have been described elsewhere [28]. Eligible individuals self-identified as Puerto Rican by birth or parentage, lived in Puerto Rico, had access to a telephone, were able to provide informed consent, and had no cognitive, psychiatric, or physical conditions limiting participation [28]. Individuals on active military duty or with an immediate family already enrolled were excluded. Data collection involved a baseline questionnaire, food frequency questionnaire, and clinical assessments. Trained staff conducted recruitment, survey administration, in-person physical examinations, and laboratory testing. The University of Puerto Rico-Medical Sciences Campus Institutional Review Board approved the study (protocol number 2290033724R001), and all participants provided written informed consent.

### Study participants

Of the 3,050 participants enrolled in the PR-OUTLOOK cohort (2020–2023), individuals were excluded from this analysis if they were missing demographic data (n = 245), individual-level SDOH variable responses (n = 10), high-risk behavior variable responses (use of cigarette smoking, electronic cigarette (e-cigarette), and recreational marijuana) (n = 3), or covariate data (n = 65). Approximately 10% of participants had missing alcohol data. To preserve sample size and reduce bias, multivariate imputation by chained equations (MICE) was applied to the alcohol variable only. The final analytic sample consisted of 2,727 participants.

### Individual-level SDOH score

The primary exposure variable was a composite individual-level SDOH score based on the Healthy People 2030 SDOH framework [5], which comprises five domains: economic stability, social connectedness, neighborhood and built environment, educational access and quality, and healthcare access and quality. Ten variables from the baseline survey data were selected, including difficulty making ends meet, food insecurity, and housing instability (economic stability); frequency of contact with family and friends (social connectedness); perceptions of neighborhood helpfulness, trust, and interpersonal relationships (neighborhood cohesion); highest educational attainment (education); and affordability of needed medical care (healthcare access).

To represent these domains, questions included: “How difficult or easy is it currently for your household to make ends meet?”, “Have you ever experienced a time when you had no place to live?”, “How often are you in contact with any of your friends?”, What is your highest level of education?”, “Was there a time in the past 12 months when you needed to see a doctor but could not because of cost?” The full list of questions corresponding to each SDOH domain is provided in Supplementary Material Table S1.

Variables were retained in their original form (binary or ordinal) to preserve their structure and minimize information loss. A polychoric correlation matrix was estimated, followed by principal factor analysis, to extract a single latent SDOH factor. Regression-based factor scoring generated a continuous index (individual-level SDOH score), with higher values reflecting greater social disadvantage. The final factor score was approximately normally distributed and modeled both continuously and in sample-based tertiles (least, moderate, and most disadvantaged). Latent factor analysis statistics are presented in Supplementary Material Table S2.

### Neighborhood-level SDOH

Neighborhood-level SDOH were assessed using the SVI developed by the Centers for Disease Control and Prevention and the Agency for Toxic Substances and Disease Registry [30]. The SVI includes 16 indicators across four domains: socioeconomic status, household characteristics, minority status and language, and housing and transportation [30]. Scores range from 0 to 1, with higher values indicating greater vulnerability. Participants’ residential address data were geocoded to 2020 U.S. Census Tracts and linked to the overall and domain-specific SVI scores, which were modeled continuously.

### High-risk behaviors

Five binary high-risk behaviors were examined: cigarette smoking, e-cigarette use, recreational marijuana use, binge drinking, and physical inactivity. Lifetime cigarette smoking was defined using the question, “Have you ever smoked at least 100 cigarettes in your entire life?” Responses were coded as a binary variable (no/yes), with “yes” indicating lifetime smoking. E-cigarette use was assessed by asking, “Have you ever smoked an electronic cigarette (e-cigarette) or vaper, ever once?” This was similarly coded as a binary variable (no/yes). Recreational marijuana use was determined based on responses to the question, “Have you ever smoked marijuana, Krispy, or hashish?” and coded as a binary indicator of any use (no/yes). Binge drinking was defined using the American Heart Association scientific statement in conjunction with alcohol drinking patterns determined by the National Institute of Alcohol Abuse and Alcoholism [24, 31], based on average daily alcohol intake derived from a validated, self-reported food frequency questionnaire. MICE was only used to impute missing self-reported alcohol data. The imputation model included age, sex, marital status, vaping and cigarette smoking frequency, and physical activity indicators. Full details of the procedure are provided in the Additional file: MICE Methodology. We first constructed a categorical variable to capture daily alcohol drinking levels: low-risk (males: ≤28 g/day, females: ≤14 g/day), moderate-risk (males: >28 - <70 g/day, females: >14 - <56 g/day), and high-risk (binge drinking) (males: ≥70, females: ≥56). Binge drinking was defined as ≥ 70 grams/day for men and ≥ 56 grams/day for women, approximating five or more drinks per occasion for males and four or more drinks per occasion for females. A binary indicator was created to reflect binge-level alcohol use versus non-binge-level use. Physical inactivity was assessed using self-reported frequency and duration of walking and mild, moderate, and strenuous physical activity. Moderate-to-vigorous physical activity (MVPA) was computed as the sum of walking, moderate, and strenuous metabolic equivalent of task (MET) in minutes/week and dichotomized as activity (≥ 500 MET-min/week) versus insufficiently active/inactive (< 500 MET-min/week) according to the Physical Activity Guidelines for Americans [32]. A count variable (0–5) summed the number of high-risk behaviors.

### Covariates

Covariates included age, sex at birth (male/female), marital status, and household income, all collected through standardized self-report questions. Marital status was categorized as single/never married, married/living with partner, and separated/divorced/widowed. Household income ranges were categorized as <$20,000, $20,000-$49,999, $50,000-$79,999, ≥$80,000, or unknown. Age, sex, and marital status were used in adjusted models for individual-level SDOH, and age (centered at the mean) and sex were used in adjusted models for fixed-effect models that included SVI (neighborhood-level contextual model). Age was centered in neighborhood-level fixed-effects models to facilitate the interpretation of the intercept.

### Statistical analysis

Descriptive statistics summarized participant characteristics across individual-level SDOH tertiles and sex. Continuous variables were reported as means and standard deviations (SD), and categorical variables as frequencies and percentages. The total count of high-risk behaviors was cross-tabulated by SDOH tertiles to describe the distribution of risk behaviors in the overall analytic sample and stratified by sex. Differences were assessed using chi-square tests for categorical variables, analyses of variance for continuous variables, and Cuzick’s non-parametric test for trend to assess monotonic trends in high-risk behaviors across ordered SDOH tertiles, stratified by sex.

Adjusted prevalence ratios (aPRs) comparing the most versus the least SDOH disadvantaged groups were estimated using Poisson regression with robust standard errors, adjusting for sex, age, and marital status. Sex-stratified analyses were used to assess whether the association between individual-level SDOH tertiles and binary high-risk behavior outcomes differed by sex at birth. Additionally, Poisson regression with robust standard errors was used to assess the cumulative association between individual-level SDOH tertiles and the total count of high-risk behaviors.

Neighborhood-level associations were estimated using fixed-effects logistic regression with census tract-level cluster-robust standard errors. Multilevel random-intercept models were explored; however, intraclass correlation coefficients indicated minimal between-tract variance, and multilevel models were not retained. Adjusted odds ratios (aORs) comparing the most versus least disadvantaged individual-level SDOH tertiles and per-unit increases in continuous SVI were estimated. Post-estimation predictive margins on the log-odds scale were used to estimate adjusted outcomes values across SDOH tertiles, allowing for the assessment of the direction, magnitude, and consistency of associations across levels of social disadvantage and confirming trends observed in the main fixed-effects logistic regression models. All analyses were conducted in Stata MP version 18.0 (StataCorp, College Station, TX). Significant differences were considered at a two-tailed p < 0.05.

## Results

Participants had a mean age of 22.6 years (SD = 3.1), and 61.2% were female, most were single/never married (86.2%), and educational attainment was split primarily between college graduates (41.1%) and those with high school degree or equivalent level (34.6%) (Table 1). Most participants reported annual household incomes under $50,000, with 29.6% earning under $20,000. Lifetime cigarette smoking (10.2%), e-cigarette use (42.9%), recreational marijuana use (45.1%), and current physical inactivity (48.2%) behavior engagement were more prevalent among those in the most SDOH disadvantaged tertiles than among the least and moderate SDOH tertiles. Overall, the mean individual-level SDOH score was 1.67 (SD = 0.65; range: 0.98 to 2.41), and the mean census tract-level SVI score was 0.45 (SD = 0.29; range: 0.44 to 0.46).

Overall, 33.7% of participants reported one high-risk behavior, 25.1% reported two, and 18.5% reported three or more (Table 2). In sex-stratified analyses, parametric test indicated a statistically significant trend toward higher high-risk behavior counts across increasing SDOH tertiles in both males (p = 0.0191) and females (p = 0.0119), although category-specific distributions were not strictly monotonic. High-risk behavior frequency of engagement is presented in Supplementary Material Table S3.

Table 1. Characteristics of Resident Puerto Rican Young Adults (Ages 18–29 Years), PR-OUTLOOK Study (n = 2,727).

Table 2. Prevalence of High-Risk Behavior Counts and SDOH Tertile by Sex, PR-OUTLOOK Study (n = 2,727).

Table 3 represents the results from the Poisson regression with robust standard error models assessing the cumulative association between individual-level SDOH disadvantage tertiles and high-risk behavior count. Compared with individuals in the least disadvantaged tertile, individuals in the most disadvantaged tertile had a 12% higher rate of engaging in multiple high-risk behaviors (aPR: 1.12, 95% CI: 1.04–1.20), adjusting for age, sex, and marital status.

Table 4 shows the associations between individual-level SDOH disadvantage and each specific high-risk behavior. Compared with participants in the least SDOH disadvantaged tertile, those in the most disadvantaged tertile had a 71% higher prevalence of cigarette smoking (aPR: 1.71, 95% CI: 1.24–2.35) and a 24% higher prevalence of physical inactivity (aPR: 1.24, 95% CI: 1.11–1.38), adjusting for age, sex, and marital status.

Table 4. Associations Between Individual-Level SDOH Tertiles and High-Risk Behaviors, PR-OUTLOOK Study (n = 2,727).

### Individual-level SDOH sex differences

Table 5 displays the results of sex-stratified Poisson regression models. Among males, those in the most disadvantaged tertile had a 41% higher prevalence of physical inactivity compared with the least disadvantaged tertile (aPR: 1.41, 95% CI: 1.16–1.72). Among females, those in the most disadvantaged tertile had more than twice the prevalence of cigarette smoking (aPR: 2.29, 95% CI: 1.39–3.79), as well as higher prevalence of e-cigarette use (aPR: 1.18, 95% CI: 1.01–1.13) and physical inactivity (aPR: 1.17, 95% CI: 1.03–1.33), compared with those in the least disadvantaged tertile.

Table 5. Associations Between Individual-Level SDOH Tertiles and High-Risk Behaviors by Sex, PR-OUTLOOK Study (n = 2,727).

### Individual-level SDOH and SVI models

Individuals in the most disadvantaged individual-level SDOH tertile had significantly higher odds of cigarette smoking (aOR: 1.91, 95% CI: 1.33–2.73), e-cigarette use (aOR: 1.23, 95% CI: 1.01–1.49), and physical inactivity (aOR: 1.46, 95% CI: 1.21–1.76) compared with those in the least disadvantaged tertile (Table 6). No significant associations were observed for the moderate SDOH tertile in the adjusted models. Neighborhood-level disadvantage, measured by census tract-level SVI, was independently associated with lower odds of e-cigarette use (aOR: 0.76, 95% CI: 0.58–1.00) and recreational marijuana use (aOR: 0.68, 95% CI: 0.51–0.92) and with increased odds of binge drinking (aOR: 1.85, 95% CI: 1.04–3.31). SVI was not significantly associated with cigarette smoking or physical inactivity. Post-estimation margin analyses indicated that log odds increased (became less negative) with increasing levels of disadvantage across all high-risk behaviors, corresponding to higher predicted probabilities of engaging in these behaviors among more socially disadvantaged tertiles (Supplementary Material Table S4).

Table 6. Associations of Individual-Level SDOH Tertiles and Neighborhood-Level SVI with High-Risk Behaviors, PR-OUTLOOK Study (n = 2,727).

### Individual-level SDOH and SVI models sex differences

In sex-stratified models, individual-level social disadvantage was more consistently associated with high-risk behaviors among females than among males (Table 7). Among females, those in the most disadvantaged tertile had significantly higher odds of cigarette smoking (aOR: 2.45, 95% CI: 1.42–4.22), e-cigarette use (aOR: 1.33, 95% CI: 1.02–1.73), and physical inactivity (aOR: 1.36, 95% CI: 1.08–1.70), compared with those in the least disadvantaged tertile. In contrast, among males, the most disadvantaged tertile had higher odds of physical inactivity (aOR: 1.68, 95% CI: 1.22–2.31), with no statistically significant associations for the other behaviors. Neighborhood-level disadvantage was also significantly associated with higher odds of binge drinking among males (aOR: 2.71, 95% CI: 1.19–6.19) and lower odds of recreational marijuana use among females (aOR: 0.69, 95% CI: 0.47–0.99).

Table 7. Associations of Individual-Level SDOH Tertiles and Neighborhood-Level SVI with High-Risk Behaviors, Stratified by Sex, PR-OUTLOOK.

## Discussion

In this cohort of young Puerto Rican adults, individual-level social disadvantage was consistently associated with greater odds of engaging in several distinct high-risk behaviors, including cigarette smoking, e-cigarette use, and physical inactivity. Contextual disadvantage, measured by the SVI, was independently associated with several behaviors, most notably binge drinking and recreational marijuana use. These associations were more pronounced among females than among males suggesting sex-specific patterns of vulnerability to social disadvantage which may shape health-related behaviors.

These findings align with a substantial body of literature linking social disadvantage to increased health-related risk behaviors among young and emerging adults. Nationally, lifestyle risk factors such as smoking, excessive alcohol use, and physical inactivity have been shown to follow clear socioeconomic gradients [33]. Our results reinforce a similar pattern for young adults in Puerto Rico, with individuals in the most disadvantaged tertile of individual-level SDOH experiencing significantly higher odds of engaging in several high-risk behaviors.

However, few studies have examined these patterns specifically among young adults residing in Puerto Rico, particularly within the context of considering both individual and contextual social determinants. Our findings add to the literature by demonstrating that patterns of disadvantage (social gradients) and associated behavioral risks among Puerto Rican young adults resemble those observed in the broader U.S. young adult population, while also reflecting distinct local district characteristics of the local territory context. Importantly, the behavior risk profiles observed in our cohort were largely consistent with 2020–2021 Puerto Rico BRFSS estimates for similar age groups. However, e-cigarette use was substantially more prevalent in our cohort (41%) compared with BRFSS estimates among individuals aged 18–24 years (4.9%) and 25–34 years (3.6%) in 2021[20]. In contrast, compared with 2020 BRFSS estimates, cigarette smoking (7.5% in our cohort vs. 6.6% among 18–24 years and 11% among 25–34 years), binge drinking (7% vs. 14.4% among 18–24 years and 14.8% among 25–34 years), and physical inactivity (42% vs. 36.1% among 18–24 years and 37.2% in 25–34 years) were slightly less prevalent in our sample [20]. These differences may suggest possible emerging generational trends in substance use or variation in measurement approaches across surveys.

Although not directly comparable by age, data from the Consulta Juvenil (2018–2020), a school-based survey of students in grades 7–12, reported ever-use prevalence of 13% for marijuana, 42% for alcohol, and 22% for e-cigarettes among Puerto Rican adolescents [34]. Whereas in separate study using 2017–2019 BRFSS using primarily states within the continental U.S., marijuana use has been reported more frequently among young adult males (aged 18–24 years) and individuals with higher socioeconomic status [35]. Although these findings arise from somewhat differing populations and age groups, they underscore the importance of understanding behavior trajectories across developmental stages and social contexts and demonstrate the need for ongoing surveillance of health-related risk behaviors on the island.

### Individual-level SDOH

Individual-level SDOH disadvantage was strongly associated with multiple high-risk behaviors. Participants in the most disadvantaged tertile had substantially higher likelihood of cigarette smoking and physical inactivity, even after accounting for age, sex, and marital status. These findings are consistent with prior evidence linking lower individual socioeconomic status (SES) to elevated health-related behavior risk among emerging adults. Structural factors, including limited access to recreational resources, heightened exposure to targeted marketing of tobacco products [36, 37], and chronic psychosocial stress [25], may help explain these patterns.

In Puerto Rico, these associations may be further exacerbated by long-standing structural stressors, including persistent economic precarity [38, 39], compounded by the impacts of Hurricanes Irma and María [9], and the global coronavirus disease 2019 (COVID-19) pandemic, which disrupted social services and strained the health system [8, 10, 11, 36]. Although these events affected the island broadly, their consequences disproportionally burdened individuals with fewer socioeconomic resources. Together, these events may intensify maladaptive coping behaviors, reduce opportunities for healthpromoting activities, and contribute to the clustering of risk factors observed in this cohort [40, 41].

Our results are consistent with social epidemiological theories, including the Fundamental Cause Theory, which argues that SES persistently shapes health outcomes through multiple, flexible mechanisms, such as differential access to resources and the ability to avoid risks or adopt protective strategies [25]. Similar associations have been observed in studies of Latinx populations in the mainland U.S., where lower individual SES is associated with increased tobacco use [13], physical inactivity [14], and poorer diet quality [15]. Studies in Puerto Rico among older adults (21–79 years) also documented strong associations between lower individual SES and elevated risk of tobacco use, physical inactivity, excessive alcohol intake, and poorer diet quality [17, 42]. Overall, these findings reinforce the value of addressing upstream social conditions, such as education, employment, and community resources, in shaping health beyond individual behaviors. For young adults in Puerto Rico, health equity efforts must account for both enduring socioeconomic disadvantage and the compounded structural stressors specific to the island’s social and political context.

### Contextual disadvantage

Neighborhood-level social disadvantage showed a complex pattern of associations with high-risk health behaviors. Unlike individual-level SDOH, which was positively associated with cigarette smoking, e-cigarette use, and physical inactivity, SVI was not consistently associated with elevated odds across behaviors. In adjusted models, higher tract-level SVI was associated with greater odds of binge drinking, but lower odds of e-cigarette use and recreational marijuana use, with no significant associations for cigarette smoking or physical inactivity. This mixed pattern suggests that contextual disadvantage may influence risk behaviors through pathways that do not always align with traditional socioeconomic gradients.

Several mechanisms may help explain these findings. Young adults living in higher-vulnerability neighborhoods may experience greater economic constraints, fewer outlets for nicotine or marijuana products, and stronger community norms discouraging certain behaviors [43]. These structural constraints may reduce access to higher-cost products such as nicotine delivery systems or marijuana vaping devices. BRFSS analyses support this interpretation, showing that cannabis vaping was disproportionately concentrated among younger, higher-income, college-educated individuals, groups that are less likely to reside in high-SVI areas [20, 43]. Thus, the inverse association we observed between SVI and e-cigarette or marijuana use may reflect the broader social patterning of “newer” substance use behaviors among more socioeconomically advantaged young people, even within the Puerto Rican context.

In contrast, the positive association between neighborhood disadvantage and binge drinking may reflect the influence of community stressors, alcohol outlet density, or neighborhood coping cultures. Such patterns were previously documented in other U.S.-based studies of structural disadvantage and alcohol-related harm [44]. This suggests that different types of risk behaviors may respond to distinct contextual pathways.

Our findings also align with recent work that examined Puerto Rican young adults residing in Puerto Rico and the South Bronx [19]. Although their outcomes focused on cardiovascular health metrics rather than strict behaviors, they similarly identified context-dependent differences in health risk, emphasizing that early adult health is sensitive to sociopolitical and environmental conditions [19]. Taken together, these results underscore that neighborhood context is an important, but behavior-specific, determinant of health among Puerto Rican young adults.

### Sex-stratified findings

Sex-stratified analyses revealed that associations between social disadvantage and high-risk behaviors were more consistently observed among Puerto Rican females. Compared with males, females in the most disadvantaged tertile had higher odds of cigarette smoking, e-cigarette use, and physical inactivity. In contrast, among males, only physical inactivity was associated with SDOH disadvantage. These findings suggest that there is differential vulnerability among male and female young adults that is shaped by structural inequities and sociocultural context.

Within the broader literature, females often experience and internalize structural disadvantage differently than males due to variations in social roles [45], caregiving responsibilities [46], and coping mechanisms [16, 26, 27, 41]. In Puerto Rico, long-standing sex-based disparities have also been documented. For instance, U.S. census data through 2021 indicate that although females in Puerto Rico often attain higher levels of education than males, they tend to report lower household incomes [47–49]. Similar patterns were also noted in a convenience sample of Puerto Rican adults aged 30–75 years recruited from a clinic setting, where females had higher educational attainment than males, but no significant differences were found in other socioeconomic factors, including income [17].

While studies of Puerto Rican populations in the mainland U.S. have documented health disadvantages shaped by socioeconomic and neighborhood context [19], few have explicitly examined sex differences in health-related risk behaviors among Puerto Rican young adults. Our findings address this gap by showing that the social gradient in high-risk behaviors in Puerto Rico disproportionately burdens young females more than males, and reflects the ongoing environmental stressors, economic marginalization, ongoing disaster recovery, and limited health infrastructure.

Following Hurricane María and during the COVID-19 pandemic, incidents of interpersonal violence involving female victims rose [50, 51], prompting the government to declare a state of emergency in 2021 [51]. Among females, exposure to violence and economic instability may elevate psychosocial stress, reduce perceived safety, and reduce access to supportive health resources. These forces may exacerbate stress-based behaviors such as smoking or physical inactivity, deepening the impact of cumulative social disadvantage. Together, these findings support that sex differences in high-risk behaviors among this cohort of Puerto Rican young adults may not solely be attributable to individual choices but may reflect broader structural and sex-based social disadvantage.

### Limitations

Our study also has several limitations. The cross-sectional nature of the data limits causal inference. Although associations were observed between social disadvantage and health behaviors, the temporal direction of these relationships cannot be definitively determined. High-risk behavior outcomes were self-reported and therefore subject to recall and social desirability bias, which may differentially affect responses across social groups. Additionally, although the SVI is widely used, it was originally developed for disaster preparedness and may not capture the full range of neighborhood-level factors relevant to health behaviors. As others have noted, census-based proxies of neighborhood context can obscure distinctions between individual and contextual effects on health outcomes [1]. Finally, although participants were recruited from across Puerto Rico, the cohort may not fully represent the island’s young adult population aged 18–29. The overrepresentation of females, in particular, may limit the generalization of the findings to all young adults living in Puerto Rico, as well as to Puerto Ricans living on the mainland and to other Latino populations.

Our study has several strengths. First, it draws on data from a large cohort of Puerto Rican young adults, a group that is historically underrepresented in social and health research. By focusing on young adulthood, a critical development period, the analysis captures early-life patterns of risk accumulation that may shape long-term health trajectories. Second, the use of both individual- and neighborhood-level indicators of social disadvantage allows for a more nuanced examination of the multiple levels at which social determinants shape behavior. The use of robust statistical methods strengthens the validity and interpretability of the findings. Finally, multiple imputation techniques were used to address missing data, minimizing potential biases.

## Conclusions

Individual- and neighborhood-level social disadvantage were associated with multiple health-risk behaviors among Puerto Rican young adults, with associations more consistently observed among females in sex-stratified analysis. These findings highlight the potential value of integrated approaches that address educational, economic stability, and neighborhood resources. Monitoring neighborhood vulnerability may also provide an important lens for public health planning in Puerto Rico. Longitudinal studies are needed to clarify causal pathways and inform multi-level interventions aimed at reducing health-related behavior risk disparities in this population.

## Supplementary Material

Supplementary Files

This is a list of supplementary files associated with this preprint. Click to download.

- AdditionalFileTable5SDOHandHighriskbehaviorsofPROUTLOOK.docx
- AdditionalFileTable7SDOHandHighriskbehaviorsofPROUTLOOK.docx
- SuppMaterialSDOHandHighriskbehaviorsofPROUTLOOK.docx

## Figures and Tables

**Table 1:** Characteristics of Resident Puerto Rican Young Adults (Ages 18–29 Years), PR-OUTLOOK Study (n = 2,727).

Characteristics^a^	Least SDOH disadvantage (n = 909)	Moderate SDOH disadvantage (n = 909)	Most SDOH disadvantage (n = 909)	Total analytic aample (n = 2727)
Age, years (mean, SD)	22.5 (3.1)	22.6 (3.1)	22.8 (3.2)	22.6 (3.1)
Female (n, *%)*	522 (57.4)	555 (61.1)	592 (65.1)	1669 (61.2)
Marital status (n, %)				
Single	816 (89.8)	784 (86.2)	752 (82.7)	2352 (86.2)
Married/Living with Partner	92 (10.1)	120 (13.2)	147 (16.2)	359 (13.2)
Separated/Divorced/Widowed	1 (0.1)	5 (0.6)	10 (1.1)	16 (0.6)
Education (n, %)				
≥College graduate	433 (47.6)	380 (41.8)	309 (34.0)	1122 (41.1)
Some college or AA degree	157 (17.3)	205 (22.5)	263 (28.9)	625 (22.9)
High school diploma/GED	311 (34.2)	321 (35.3)	311 (33.4)	943 (34.6)
<High School diploma	8 (0.9)	3 (0.3)	26 (2.8)	37 (1.4)
Household income (n, %)				
$0–$19,999	202 (22.2)	237 (26.1)	369 (40.6)	808 (29.6)
$20,000–$49,999	251 (27.6)	295 (32.4)	231 (25.4)	777 (28.5)
$50,000–$79,999	154 (16.9)	104 (11.4)	86 (9.5)	344 (12.6)
≥$80,000	165 (18.2)	102 (11.2)	38 (4.2)	305 (11.2)
Does not know	137 (15.1)	171 (18.8)	185 (20.3)	493 (18.1)
Cigarette smoking (n, %)				
Yes	51 (5.6)	60 (6.6)	93 (10.2)	204 (7.5)
E-cigarette use (n, %)				
Yes	355 (39.1)	387 (42.6)	390 (42.9)	1132 (41.5)
Recreational marijuana use (n, %)				
Yes	407 (44.8)	413 (45.4)	411 (45.1)	1231 (45.1)
Binge drinking (n, %)				
Yes	64 (7.0)	55 (6.1)	57 (6.3)	176 (6.5)
Physical inactive (n, %)				
Yes	341 (37.5)	385 (42.4)	438 (48.2)	1164 (42.7)
Individual-level SDOH score (mean, SD)	1.00 (0.29)	1.63 (0.15)	2.38 (0.45)	1.67 (0.65)
SVI score (neighborhood-level) (mean, SD)	0.42 (0.28)	0.45 (0.29)	0.49 (0.29)	0.45 (0.29)
SVI domains (mean, SD)				
SES score	0.42 (0.28)	0.44 (0.28)	0.48 (0.28)	0.44 (0.28)
Household and disability score	0.43 (0.29)	0.45 (0.28)	0.48 (0.29)	0.45 (0.29)
Minority status and language score	0.37 (0.18)	0.38 (0.18)	0.40 (0.18)	0.38 (0.18)
Housing type and transportation score	0.47 (0.28)	0.50 (0.29)	0.52 (0.29)	0.50 (0.29)

Abbreviations: AA, associate’s degree; GED, general educational development; PR-OUTLOOK, Puerto Rico Young Adults’ Stress, Contextual, Behavioral & Cardiometabolic Risk; SD, standard deviation; SDOH, Social determinants of health; SES, socioeconomic status; SVI, Social Vulnerability Index.

a Data expressed as means (SD) and frequencies (n (%)).

**Table 2:** Prevalence of High-Risk Behavior Counts and SDOH Tertile by Sex, PR-OUTLOOK Study (n = 2,727).

High-risk behavior count^b^	Male^a^			Female^a^			Total analytic sample^a^ (n = 2727)
	Least SDOH disadvantage (n = 387)	Moderate SDOH disadvantage (n = 354)	Most SDOH disadvantage (n = 317)	Least SDOH disadvantage (n = 522)	Moderate SDOH disadvantage (n = 555)	Most SDOH disadvantage (n = 592)	
0 behaviors	93 (24.0)	76 (21.5)	60 (18.9)	133 (25.4)	122 (22.0)	135 (22.8)	619 (22.7)
1 behavior	117 (30.8)	112 (31.6)	90 (28.4)	193 (39.7)	208 (37.5)	198 (33.4)	920 (33.7)
2 behaviors	110 (28.4)	100 (28.2)	94 (26.7)	120 (23.0)	129 (23.2)	131 (22.1)	684 (25.1)
3 behaviors	50 (12.9)	52 (3.9)	57 (17.9)	69 (13.2)	75 (13.5)	102 (17.2)	405 (14.8)
4 behaviors	14 (3.6)	14 (3.9)	14 (4.4)	7 (1.3)	20 (3.6)	22 (3.7)	91 (3.4)
5 behaviors	1 (0.3)	0 (0.0)	2 (0.6)	0 (0.0)	1 (0.2)	4 (0.6)	8 (0.3)

Abbreviations: PR-OUTLOOK, Puerto Rico Young Adults’ Stress, Contextual, Behavioral & Cardiometabolic Risk; SDOH, Social determinants of health.

Cuzick’s non-parametric test for trend was used to assess monotonic trends in high-risk behavior counts across ordered SDOH tertiles, stratified by sex (p = 0.0191 for males; p = 0.0119 for females).

a Data are expressed as frequencies (n, %).

b High-risk behaviors include cigarette smoking, e-cigarette use, recreational marijuana use, binge alcohol drinking, and physical inactivity.

**Table 3:** Cumulative Association Between Individual SDOH Tertiles and High-Risk Behavior Count, PR-OUTLOOK Study (n = 2,727).^a^

	Unadjusted (PR, 95% CI)		Adjusted^b^ (aPR, 95% CI)	
	Moderate SDOH disadvantage	Most SDOH disadvantage	Moderate SDOH disadvantage	Most SDOH disadvantage
High-risk behavior count	1.06 (0.99–1.14)	1.14 (1.06–1.22)**	1.06 (0.98–1.13)	1.12 (1.04–1.20)**

Abbreviations: CI, Confidence interval; PR, Prevalence ratio; PR-OUTLOOK, Puerto Rico Young Adults’ Stress, Contextual, Behavioral & Cardiometabolic Risk; SDOH, Social determinants of health.

a Model estimates were obtained using Poisson regression with robust standard errors, comparing each SDOH tertiles with the least disadvantaged tertile. High-risk behavior count was defined as the number of the following behaviors present in each individual: cigarette smoking, e-cigarette use, recreational marijuana use, binge drinking, and physical inactivity.

b Models adjusted for age, sex, and marital status.

** p < 0.001

**Table 4:** Associations Between Individual-Level SDOH Tertiles and High-Risk Behaviors, PR-OUTLOOK Study (n = 2,727).

High-risk behaviors	Unadjusted (PR, 95% CI)^a^		Adjusted (aPR, 95% CI)^a,b^	
	Moderate SDOH disadvantage	Most SDOH disadvantage	Moderate SDOH disadvantage	Most SDOH disadvantage
Cigarette smoking	1.18 (0.82–1.68)	1.82 (1.31–2.53)***	1.15 (0.81–1.64)	1.71 (1.24–2.35)**
E-cigarette use	1.09 (0.98–1.22)	1.10 (0.98–1.23)	1.09 (0.98–1.22)	1.10 (0.99–1.23)
Recreational marijuana use	1.01 (0.92–1.12)	1.00 (0.91–1.11)	1.01 (0.91–1.12)	0.99 (0.89–1.09)
Binge drinking	0.86 (0.60–1.21)	0.89 (0.63–1.25)	0.85 (0.60–1.20)	0.88 (0.62–1.25)
Physical inactivity	1.13 (1.01–1.26)*	1.28 (1.15–1.43)***	1.11 (0.99–1.24)*	1.24 (1.11–1.38)***

Abbreviations: CI, Confidence interval; PR, Prevalence ratio; PR-OUTLOOK, Puerto Rico Young Adults’ Stress, Contextual, Behavioral & Cardiometabolic Risk; SDOH, Social determinants of health.

a Model estimates were obtained using Poisson regression with robust standard errors, comparing each SDOH tertiles with the least disadvantaged tertile.

b Models adjusted for age, sex, and marital status.

* p < 0.05

** p < 0.01

*** p < 0.001

**Table 5:** Associations Between Individual-Level SDOH Tertiles and High-Risk Behaviors Stratified by Sex, PR-OUTLOOK Study (n = 2,727).

High-risk behaviors	Males^a^				Females^a^			
	Unadjusted (PR, 95% CI)		Adjusted (aPR, 95% CI)^b^		Unadjusted (PR, 95% CI)		Adjusted (aPR, 95% CI)^b^	
	Moderate SDOH disadvantage	Most SDOH disadvantage	Moderate SDOH disadvantage	Most SDOH disadvantage	Moderate SDOH disadvantage	Most SDOH disadvantage	Moderate SDOH disadvantage	Most SDOH disadvantage
Smoking cigarettes	1.09 (0.68–1.75)	1.53 (0.98–2.37)	1.06 (0.67–1.68)	1.40 (0.91–2.15)	1.38 (0.78–2.45)	2.45 (1.47–4.09)**	1.35 (0.77–2.37)	2.29 (1.39–3.79)**
E-cigarette use	1.07 (0.93–1.25)	1.04 (0.89–1.21)	1.07 (0.92–1.24)	1.02 (0.87–1.19)	1.12 (0.96–1.32)	1.19 (1.02–1.39)*	1.12 (0.95–1.32)	1.18 (1.01–1.37)*
Recreational marijuana use	1.01 (0.88–1.16)	1.03 (0.89–1.19)	1.00 (0.87–1.15)	1.00 (0.87–1.16)	1.03 (0.90–1.19)	1.02 (0.89–1.17)	1.02 (0.88–1.16)	0.98 (0.86–1.13)
Binge drinking	0.74 (0.42–1.30)	1.05 (0.62–1.76)	0.73 (0.42–1.28)	1.04 (0.62–1.75)	0.94 (0.60–1.46)	0.81 (0.51–1.28)	0.93 (0.59–1.45)	0.79 (0.50–1.25)
Physical inactivity	1.10 (0.89–1.36)	1.41 (1.16–1.71)**	1.11 (0.89–1.37)	1.41 (1.16–1.72)**	1.12 (0.98–1.28)	1.19 (1.05–1.35)**	1.11 (0.97–1.26)	1.17 (1.03–1.33)*

Abbreviations: CI, Confidence interval; PR, Prevalence Ratio; PR-OUTLOOK, Puerto Rico Young Adults’ Stress, Contextual, Behavioral & Cardiometabolic Risk; SDOH, Social determinants of health.

a Model estimates were obtained using Poisson regression with robust standard errors, stratified by sex, comparing each SDOH tertile with the least disadvantaged tertile.

b Models adjusted for age and marital status.

* p < 0.05

** p < 0.01

**Table 6:** Associations of Individual-Level SDOH Tertiles and Neighborhood-Level SVI With High-Risk Behaviors, PR-OUTLOOK Study (n = 2,727).

High-risk behaviors	Unadjusted (OR, 95% CI)^a^			Adjusted (aOR, 95% CI)^a,b^		
	Moderate SDOH disadvantage	Most SDOH disadvantage	Overall SVI^c^	Moderate SDOH disadvantage	Most SDOH disadvantage	Overall SVI^c^
Cigarette smoking	1.18 (0.80–1.73)	1.89 (1.33–2.68)**	1.19 (0.73–1.96)	1.19 (0.96–1.41)	1.91 (1.33–2.73)**	1.16 (0.69–1.92)
E-cigarette use	1.16 (0.96–1.41)	1.19 (0.98–1.45)	0.75 (0.57–0.98)*	1.18 (0.98–1.43)	1.23 (1.01–1.49)*	0.76 (0.58–1.00)
Recreational marijuana use	1.03 (0.86–1.25)	1.04 (0.86–1.26)	0.70 (0.52–0.94)*	1.04 (0.86–1.26)	1.03 (0.85–1.25)	0.68 (0.51–0.92)*
Binge drinking	0.83 (0.57–1.22)	0.84 (0.58–1.21)	1.85 (1.04–3.31)*	0.84 (0.57–1.23)	0.87 (0.60–1.25)	1.85 (1.04–3.31)*
Physical inactivity	1.21 (1.01–1.46)*	1.52 (1.26–1.83)*	1.28 (0.98–1.66)	1.19 (0.98–1.44)	1.46 (1.21–1.76)**	1.24 (0.95–1.61)

Abbreviations: CI, confidence interval; OR, Odds Ratio; PR-OUTLOOK, Puerto Rico Young Adults’ Stress, Contextual, Behavioral & Cardiometabolic Risk; SDOH, Social determinants of health; SVI, Social Vulnerability Index.

a Model estimates were obtained using fixed-effect logistic regression with cluster-robust standard errors by census tract (2020), comparing each SDOH tertile with the least disadvantaged tertile.

b Models adjusted for age and sex (centered at the mean).

c SVI represents contextual social disadvantage at the census tract level, derived from the 2020 CDC/ATSDR composite SVI score linked to U.S. census data.

* p < 0.05

** p < 0.001

**Table 7:** Associations of Individual-Level SDOH Tertiles and Neighborhood-Level SVI With High-Risk Behaviors, Stratified by Sex, PR-OUTLOOK.

High-risk behaviors	Models^a,b^ (OR, 95% CI)	Males			Females		
		Moderate SDOH disadvantage	Most SDOH disadvantage	Overall SVI^c^	Moderate SDOH disadvantage	Most SDOH disadvantage	Overall SVI^c^
Cigarette smoking	Unadjusted	1.10 (0.66–1.80)	1.58 (0.97–2.58)	1.10 (0.60–2.04)	1.39 (0.76–2.51)	2.55 (1.50–4.34)**	1.40 (0.66–2.93)
	Adjusted	1.09 (0.65–1.82)	1.52 (0.92–2.52)	0.97 (0.50–1.87)	1.36 (0.74–2.50)	2.45 (1.42–4.22)**	1.38 (0.65–2.92)
E-cigarette use	Unadjusted	1.16 (0.86–1.55)	1.10 (0.82–1.48)	0.73 (0.47–1.12)	1.21 (0.93–1.57)	1.34 (1.03–1.74)*	0.80 (0.56–1.13)
	Adjusted	1.16 (0.86–1.55)	1.09 (0.81–1.46)	0.71 (0.46–1.09)	1.21 (0.93–1.56)	1.33 (1.02–1.73)*	0.80 (0.56–1.13)
Recreational marijuana use	Unadjusted	1.02 (0.76–1.36)	1.09 (0.81–1.47)	0.73 (0.48–1.13)	1.06 (0.83–1.37)	1.05 (0.83–1.35)	0.70 (0.49–1.00)
	Adjusted	1.03 (0.76–1.39)	1.05 (0.77–1.44)	0.67 (0.43–1.04)	1.05 (0.81–1.36)	1.02 (0.79–1.30)	0.69 (0.47–0.99)*
Binge drinking	Unadjusted	0.70 (0.37–1.32)	0.95 (0.53–1.71)	2.61 (1.15–5.94)*	0.92 (0.57–1.49)	0.77 (0.47–1.27)	1.85 (0.46–3.12)
	Adjusted	0.70 (0.37–1.32)	0.97 (0.54–1.74)	2.71 (1.19–6.19)*	0.93 (0.57–1.51)	0.79 (0.48–1.31)	1.46 (0.68–3.14)
Physical inactivity	Unadjusted	1.15 (0.83–1.58)	1.69 (1.23–2.32)**	1.10 (0.71–1.70)	1.22 (0.95–1.57)	1.37 (1.09–1.72)**	1.31 (0.94–1.84)
	Adjusted	1.15 (0.83–1.58)	1.68 (1.22–2.31)**	1.09 (0.70–1.70)	1.21 (0.94–1.56)	1.36 (1.08–1.70)**	1.31 (0.94–1.85)

Abbreviations: CI, Confidence interval; OR, Odds ratio; PR-OUTLOOK, Puerto Rico Young Adults’ Stress, Contextual, Behavioral & Cardiometabolic Risk; SDOH, Social determinants of health; SVI, Social Vulnerability Index.

a Model estimates were obtained using fixed-effect logistic regression with cluster-robust standard errors by census tract (2020), comparing each SDOH tertile with the least disadvantaged tertile, stratified by sex. Includes 2727 participants.

b Models adjusted for age (centered at the mean).

c SVI represents contextual social disadvantage at the census tract level, derived from the 2020 CDC/ATSDR composite SVI score linked to U.S. census data.

* p < 0.05

** p < 0.01

## Data Availability

The datasets generated and analyzed during the current study are not publicly available due to the corresponding author not having permission to share the data. However, PR-OUTLOOK data, including the datasets generated and analyzed in the present study, are available through a manuscript proposal process through the PR-OUTLOOK Primary Investigators and Review Committee.

## Abbreviations

AA: Associate’s degree
ATSDR: Agency for Toxic Substances and Disease Registry
BRFSS: Behavioral Risk Factor Surveillance System
CI: Confidence interval
CDC: Centers for Disease Control and Prevention
COVID-19: Global coronavirus disease 2019
CVD: Cardiovascular disease
GED: General educational development
MET: Metabolic equivalent of task
MICE: Multivariate imputation by chained equations
MVPA: Moderate-to-vigorous physical activity
OR: Odds ratio
PR-OUTLOOK: Puerto Rico Young Adults’ Stress, Contextual, Behavioral & Cardiometabolic Risk
PR: Prevalence ratio
SD: Standard deviation
SDOH: Social determinants of health
SES: Socioeconomic status
SVI: Social Vulnerability Index
U.S.: United States
